# Non-Invasive Delivery of dsRNA into De-Waxed Tick Eggs by Electroporation

**DOI:** 10.1371/journal.pone.0130008

**Published:** 2015-06-19

**Authors:** Newton Ruiz, Leonardo Araujo de Abreu, Luís Fernando Parizi, Tae Kwon Kim, Albert Mulenga, Gloria Regina Cardoso Braz, Itabajara da Silva Vaz, Carlos Logullo

**Affiliations:** 1 Unidade de Experimentação Animal and Laboratório de Química e Função de Proteínas—Universidade Estadual Norte Fluminense–Darcy Ribeiro, Campos dos Goytacazes, RJ, Brazil; 2 Laboratório Integrado de Bioquímica Hatisaburo Masuda—Núcleo em Ecologia e Desenvolvimento Sócio-Ambiental de Macaé (NUPEM/UFRJ), Macaé, RJ, Brazil; 3 Centro de Biotecnologia, Universidade Federal do Rio Grande do Sul, Porto Alegre, RS, Brazil; 4 Department of Veterinary Pathobiology, College of Veterinary Medicine, Texas A&M University, College Station, TX, United States of America; 5 Departamento de Bioquímica–Instituto de Química, Universidade Federal do Rio de Janeiro, Ilha do Fundão, RJ, Brazil; 6 Instituto Nacional de Ciência e Tecnologia em Entomologia Molecular (INCT-EM), Rio de Janeiro, Ilha do Fundão, RJ, Brazil; University of Kentucky College of Medicine, UNITED STATES

## Abstract

RNA interference-mediated gene silencing was shown to be an efficient tool for validation of targets that may become anti-tick vaccine components. Here, we demonstrate the application of this approach in the validation of components of molecular signaling cascades, such as the Protein Kinase B (AKT) / Glycogen Synthase Kinase (GSK) axis during tick embryogenesis. It was shown that heptane and hypochlorite treatment of tick eggs can remove wax, affecting corium integrity and but not embryo development. Evidence of AKT and GSK dsRNA delivery into de-waxed eggs of via electroporation is provided. Primers designed to amplify part of the dsRNA delivered into the electroporated eggs dsRNA confirmed its entry in eggs. In addition, it was shown that electroporation is able to deliver the fluorescent stain, 4',6-diamidino-2-phenylindole (DAPI). To confirm gene silencing, a second set of primers was designed outside the dsRNA sequence of target gene. In this assay, the suppression of AKT and GSK transcripts (approximately 50% reduction in both genes) was demonstrated in 7-day-old eggs. Interestingly, silencing of GSK in 7-day-old eggs caused 25% reduction in hatching. Additionally, the effect of silencing AKT and GSK on embryo energy metabolism was evaluated. As expected, knockdown of AKT, which down regulates GSK, the suppressor of glycogen synthesis, decreased glycogen content in electroporated eggs. These data demonstrate that electroporation of de-waxed *R*. *microplus* eggs could be used for gene silencing in tick embryos, and improve the knowledge about arthropod embryogenesis.

## Introduction

*Rhipicephalus (Boophilus) microplus* is an ectoparasite that affects livestock, causing economic losses and transmits important cattle disease agents, like *Babesia* spp and *Anaplasma marginale* [1]. Current acaricide-based tick control methods are effective in the short term, but do not offer a permanent solution due to serious limitations, like the selection of acaricide-resistant tick population. High fecundity allows thousands of larvae from a single female, being one of the most important factors that maintain elevated tick populations in environment. In this context, understanding the molecular basis of tick embryogenesis could be useful to aid the development of new control strategies. Considerable technological breakthroughs have led to the discovery of genes expressed during embryogenesis. However, there is a lack of tools to study the role(s) of candidate genes in embryogenesis. Current approaches to validate the significance of interest genes in tick physiology such as RNAi silencing and immunological assays are optimized mostly for the study of adult ticks to a limited extent of larva and nymph.

Conventional RNAi silencing can feasibly be applied to study embryogenesis. However, one drawback of conventional methods for double-stranded RNA (dsRNA) delivery is the potential of tick egg/embryo structure damage. A recent study explored dsRNA non-invasive delivery into unaltered *Ixodes scapularis* eggs using electroporation [2]. One advantage of electroporation in embryos is the lower damage caused, when compared to invasive delivery [3]. Electroporation is a well-established technique used to deliver DNA or dsRNA, plasmid, protein and drugs into cells [2, 4–6]. Double-stranded RNA delivery by electroporation is achieved applying high electrical voltage short pulses that increase the potential of membrane transport and promote the formation of transient aqueous pores in lipid bilayer, allowing macromolecules to migrate through these pores [7]. While electroporation enhances genetic material delivery into most cells, the tick eggs wax coat hampers dsRNA delivery into tick embryos. Egg wax is hydrophobic, which is why eggs will not completely submerge into the dsRNA solution. The wax coat may affect electrical pulses conductance onto the shell, and thus its removal may increase the rate of dsRNA passing through membrane micropores into the embryo.

In this study, we use the glucose metabolism related to cellular development [8] to evaluate the effect of silencing Protein Kinase B (AKT) and Glycogen Kinase Synthase (GSK) genes in tick embryos. During embryogenesis, the embryo uses several energy sources to modulate important metabolic pathways. Abreu and colleagues [9] demonstrated tick cell response to insulin while studying BME26 *R*. *microplus* embryonic cells line, as demonstrated by glycogen accumulation via the PI3K/AKT pathway [9]. The AKT/GSK signaling system modulates glycogen levels and other processes, such as embryonic axis formation, cell death, protein synthesis, and cell proliferation [9, 10–11]. In order to improve the knowledge about these metabolic processes, we have developed and evaluated a method to deliver dsRNA into *R*. *microplus* eggs. We describe successful de-waxing of eggs and dsRNA delivery into *R*. *microplus* eggs. Most importantly, we show that de-waxing using heptane prior to electroporation, improves gene silencing rates.

## Materials and Methods

### 2.1 Rhipicephalus (Boophilus) microplus ticks

*R*. *microplus* ticks (Porto Alegre strain) used in this study were obtained from a laboratory colony maintained on cattle at Universidade Federal do Rio Grande do Sul, Brazil. Spontaneously detached fully engorged female ticks were kept for 10 days at 28°C and 80% relative humidity, and allowed to oviposit. Seven day-old eggs denote eggs collected within the first 23 hours after oviposition and kept in incubator until the 7^th^ day after oviposition. Eggs were electroporated on the days indicated and maintained in a Petri dish for up to 28 days to determine hatching rate. Humidification by spraying of distilled water was carried out once a day, until eggs hatched. Alternatively, collected eggs were used for expression analyses at the 7^th^ seven day after the electroporation procedure.

### 2.2 Double-stranded RNA (dsRNA) synthesis

*In vitro* dsRNA synthesis was performed as previously described [9, 10]. Approximately 2 μg of the purified PCR product were synthesized using the T7 RiboMAX Express RNAi System (Promega, US) for *in vitro* dsRNA synthesis, as described [10]. The negative control (CN) for RNAi induced gene silencing was an unrelated dsRNA designed for *E*. *coli* β-galactosidase [kindly donated by Professor Marcos H. Sorgine, Instituto de Bioquímica Médica (IBqM), Universidade Federal do Rio de Janeiro (UFRJ)]. dsRNA fragments were: 635 bp for dsAKT, 800 bp for dsCN, and 798 bp for dsGSK long.

*R*. *microplus* BmAKT (accession JX648548) and BmGSK (accession EF142066) dsRNA were used as previously described [11]. Template DNA for dsRNA was amplified using RmAKT-T7F 5′-taatacgactcactatagggTCAGCCTGGACAACTTTGAGTTCCTC-3′, and RmAKT-T7R 5′-taatacgactcactatagggATTTCATACATGACCACGCCCAGC-3′, for GSK3β-T7F: 5′-taatacgactcactatagggTTATGCGACGGCTAGAACACT- 3’ and GSK3β-T7R: 5′-taatacgactcactatagggGCTCTTGCTCTGTGAAGTTGAA- 3’. The dsRNA specificity was estimated from similar genes in other species in the dsCheck and DEQOR softwares [12–13].

### 2.3 De-waxing and dsRNA delivery into eggs by electroporation

Three aliquots of eggs (150 mg each) were collected in microtubes and submitted to de-waxed treatment before electroporation was composed of three steps. In the first step, 400 μL of heptane (100%) were added and the mixture was stirred for 2 minutes, after which the liquid phase was removed. In the second step 200 μL (10%) hypochlorite were added and the mixture was gently shaken for 2 minutes. Then 200 μL of heptane (100%) were added and the biphasic mixture was stirred again for 3 minutes. After mixing the liquid phase were completely removed. Eggs were transferred to Petri dishes using 400 μL heptane (100%) using a tip with the top cut off. Immediately after heptane evaporation, each egg aliquots was covered with 100 μL of dsRNA solution (5.0 μg/μL, in 0.5 M TAE buffer pH = 7.4). Electroporation was performed using an electroporator (ECM 2001 Electro Cell Manipulation System, BTX) set to the following parameters was: 150V, 50 ms pulse-width, 10 pulses with 1-s intervals between the pulses, for each individual aliquot. After electroporation, eggs were kept for 7 days under same conditions described above. On the next day after electroporation drop containing dsRNA was removed and replaced by a drop of distilled water.

### 2.4 Gene silencing analysis using quantitative qRT-PCR

Total RNA was extracted from eggs (150 mg) using Trizol Reagent (Invitrogen) according to the manufacturer’s instructions. Two μg of total RNA were reversely transcribed with the High Capacity cDNA Reverse transcription kit (Applied Biosystems). Quantitative RT-PCR amplifications were performed using a Step One Plus platform (Applied Biosystems). cDNA serial dilutions were used to determine amplification efficiencies (85–100%) for each pair of primers in 12-μL reactions. Primers for RmAKT (accession number JX648548) [11], RmGSK3 (accession number EF142066) [14] were the same as described previously. Eggs treated with dsRNA corresponding to β galactosidase (dsβgal) were used as negative control. Ribosomal protein L4 (accession number CD794864) (RPL4) was used as reference gene for relative expression determination [15], using the Ct values from each run on the Relative Expression Software Tool—REST [16]. The capacity of electroporation to delivery dsRNA was analyzed by real-time PCR using a second pair of primers for AKT (AKTi) inside sequence for BmAKT Forward 5’ TATCTACCGGGACCTGAAGC-3’ and Reverse primer 5’ CCCGAAAG AGATGTCCTCCT 3’.

### 2.5 Confocal images to confirm the entry of DAPI probe in eggs

To evaluate the capacity of electroporation to transport molecules across the *corium*, eggs were subjected to the same de-waxing treatment used for dsRNA electroporation; however, the eggs were electropored with 400 nM DAPI (0.5 M TAE buffer pH = 7.4). After 24 hours the samples were analyzed on a LSM 710 Zeiss Axio Observer confocal microscope (Carl Zeiss AG, Germany). The images were collected using 405 nm laser for excitation and emission filters 410–585 for DAPI fluorophore. The possibility of endogenous florescence in the eggs was evaluated using treated eggs electroporated with 0.5 M TAE buffer pH = 7.4 without DAPI.

### 2.6 Glycogen content determination

Glycogen content was determined according to previous studies [8]. Briefly, egg homogenates were obtained in 200 mM acetate buffer (pH 4.8) and centrifuged at 4,000x *g*, for 6 min at 4°C. Total protein was quantified using the Bradford assay [17]. Aliquots were incubated with 1 unit of α-amyloglucosidase in 200 mM acetate buffer (pH 4.8) for 4 h at 40°C. The reaction was stopped with 100 mM phosphate buffer (pH 7.4), and glucose was enzymatically determined with a commercial kit (Glucox Doles, Goiânia, Brazil). Control conditions (without α-amyloglucosidase) were used to determine basal glucose level and subtracted from test conditions results. Glycogen content was calculated from glucose released in the medium related to a standard curve obtained under similar conditions, and normalized by total protein content. Results are shown as mean and standard deviation of three independent experiments in triplicate.

### 2.7 Hatching rate determination in AKT and GSK silenced eggs

Eggs were collected for hatching rate determination. After hatching, larvae were frozen at -20°C for 48 h prior to counting. Subsequently, hatched and unhatched eggs were counted under a stereomicroscope (Carl Zeiss AxioVision 4.8.2 SP1). The sum of hatched and unhatched eggs determined the total of number of eggs that were electroporated. This value was used to normalize the percentage of hatched eggs on each treatment condition, in three biological replicates.

### 2.6 Statistical Analysis

The non-parametric Student *t* test (*p*<0.05) qRT-PCR analysis was used to determine differences between treatment and control group analysis, for hatching rate assay was using de ANOVA and posttest Tukey (*p*<0.05) in both analyses was used the GraphPad Prism 5.03 Software (GraphPad Software, Inc.).

## Results

### 3.1 Eggs de-waxed and hatching rate

**Fig 1:**
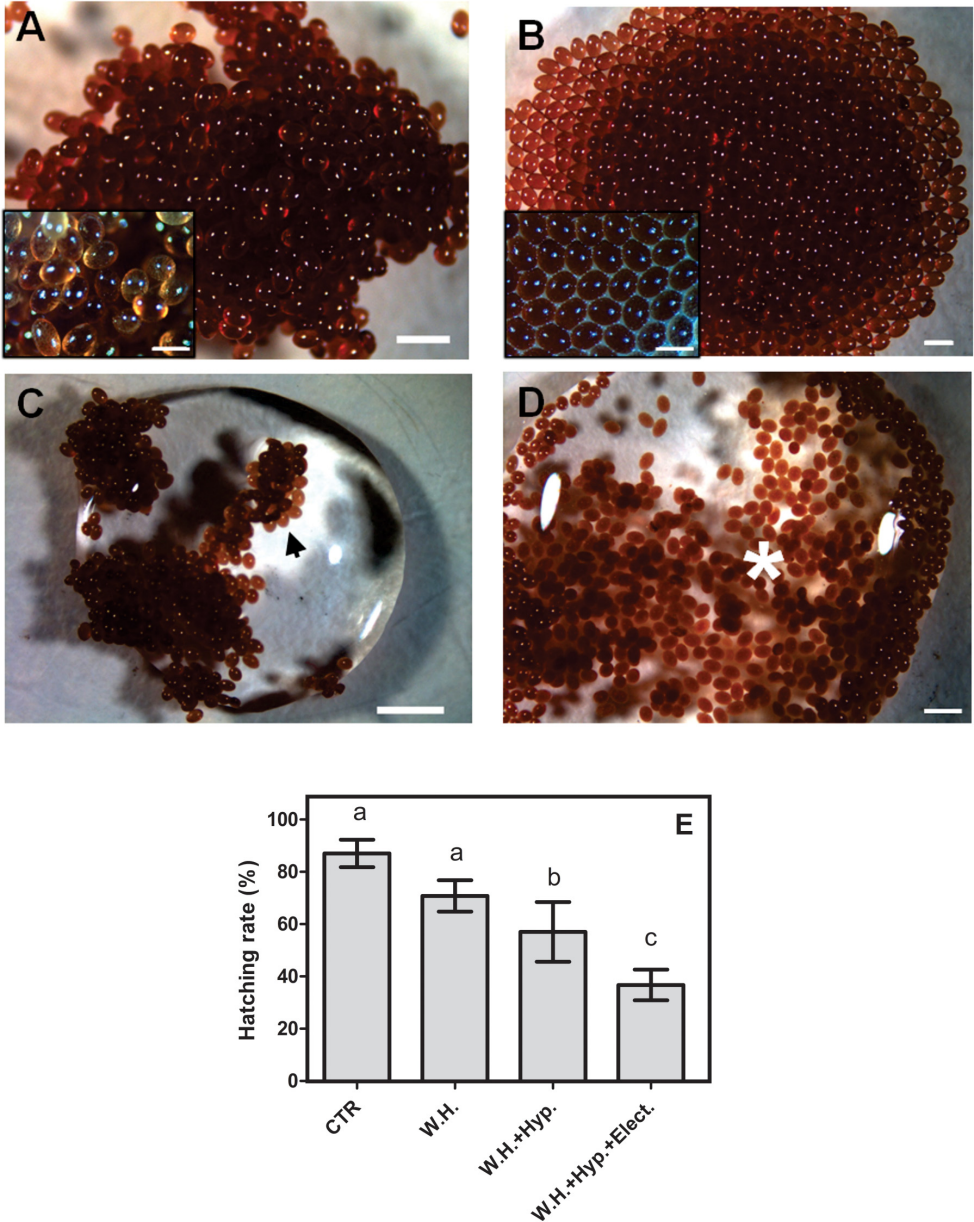
De-waxing of eggs using heptane and hypochlorite. The eggs, in different treatment, were observed using stereomicroscope as indicated in the figure. To remove the egg wax coating, 10-mg egg batches were treated with ~40–50 μL of heptane for ~15 min as described. A = unaltered egg clump, B = monolayer of treated eggs, C = clump of unaltered eggs floating on top of aqueous droplet (white arrowhead), D = de-waxed eggs submerged in the aqueous droplet (white asterisks sign). E = the hatching rate of eggs in different treatments, unaltered eggs (CTR), with heptane (W.H.), with heptane and hypochlorite (W.H. + Hyp.) and with heptane, hypochlorite and electroporated (W.H.+ Hyp.+elect). Magnification bars: A-D: 2000 μm, Insert: 500 μm. Statistical analysis was carried out using the ANOVA and posttest Tukey (*p*<0.05), (Triplicate; n = 3).

We summarize successful de-waxing of *R*. *microplus* eggs using heptane (Fig 1). Unaltered eggs clumped together (Fig 1A), while heptane treated de-waxed eggs formed a monolayer (Fig 1B). Clumped unaltered eggs floated (Fig 1C), while de-waxed eggs submerged under an aqueous droplet (Fig 1D). De-waxed eggs submerged under the water drop. To observe the effects of de-wax treatment on survival of eggs, hatching rate was determined at different steps of the treatment. It was observed that the treatment with heptane and hypochlorite affected the hatching capacity of the treated eggs by 50%, in comparison with unaltered eggs (Fig 1E and S1 Fig).

### 3.2 Confocal images to confirm the DAPI delivery by electroporation

**Fig 2:**
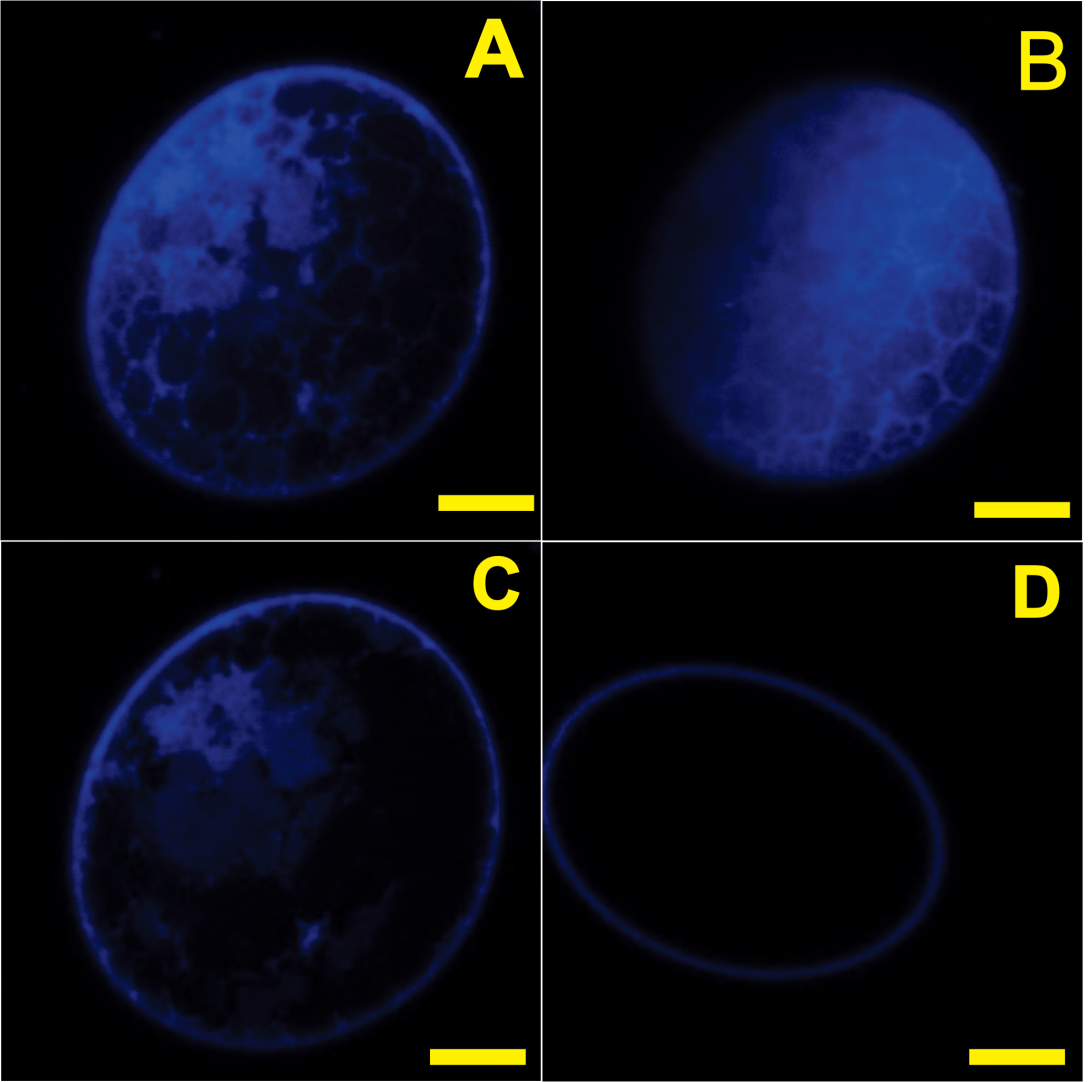
The electroporation was capable of delivering DAPI nuclear probe into the eggs. Eggs were electroporated with 0.5 M solution TAE buffer with DAPI (400 nM) and observed in confocal microscopy using 405 nm laser scanning in three different slices as shown in this figure. A) First slice in 4.81 μm, B) second slice in 8.01 μm and C) third slice in 11.22 μm, in Fig D eggs were electroporated with 0.5 M solution TAE buffer solution and observed in confocal. Magnification bars: A-D: 70 μm.

Electroporation was capable to deliver DAPI in de-waxed eggs. In the eggs electroporated with DAPI, it was possible to observe the characteristic DAPI nuclear staining in the tick embryo scanned in three different slices (Fig 2A, 2B and 2C). In the negative control (TAE solution electroporation) it was observed only the intrinsic fluorescence in the *corium* (Fig 2D).

### 3.3 qRT-PCR analysis of AKT and GSK knockdown

**Fig 3:**
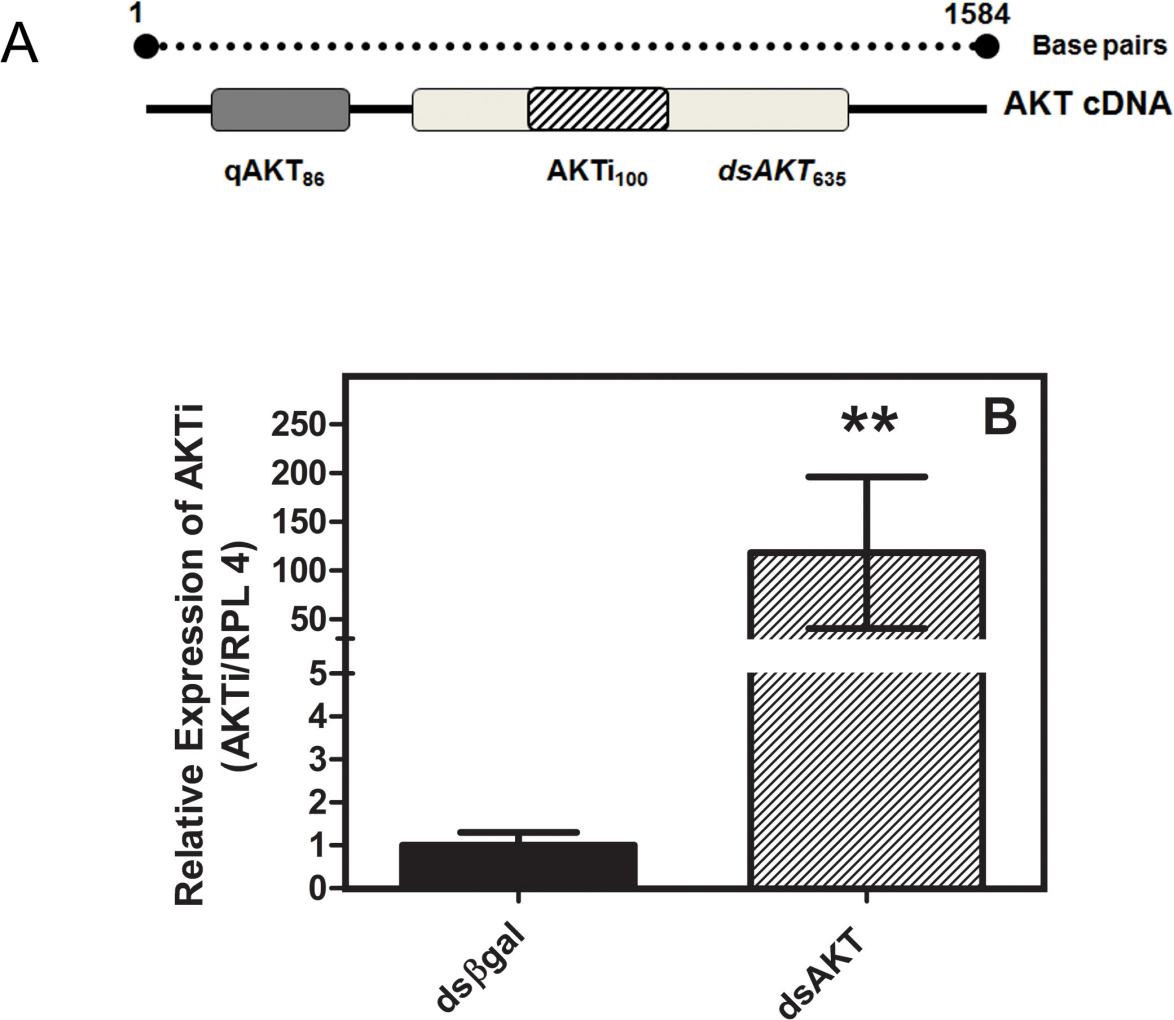
Quantitative RT-PCR validation of dsRNA delivery into *R*. *microplus* eggs by electroporation. Primers designed within the AKT dsRNA target region (3A diagonal lines = AKTi) were used to amplify AKT in control and treated eggs. A = graphical representation of the strategy to verify dsRNA delivery, gray shaded box = dsRNA region, dashed box = validation of dsRNA delivery priming site, dark gray box = validation of mRNA suppression priming site. In B the validation of dsRNA delivery in eggs were electroporated on the seventh day of embryo development, respectively. (See materials and methods). Electroporation and q-RT-PCR was performed on the days indicated. Statistical analysis was carried out using the Student t test (*p*<0.05), (Triplicate; n = 3).

To confirm dsRNA delivery into embryos, PCR primers were designed within the AKT dsRNA sequence (AKTi), as shown in Fig 3A. The delivery of dsRNA of dsRNA of AKT performed by electroporation we analyzed by real-time PCR using primers dsAKT inside the region. The transcript 120-fold higher than in the control (electroporated with unrelated dsRNA) eggs (Fig 3B), though the same amount of dsRNA was used for both conditions. These observations confirmed that AKT dsRNA delivery into the eggs was successful.

**Fig 4:**
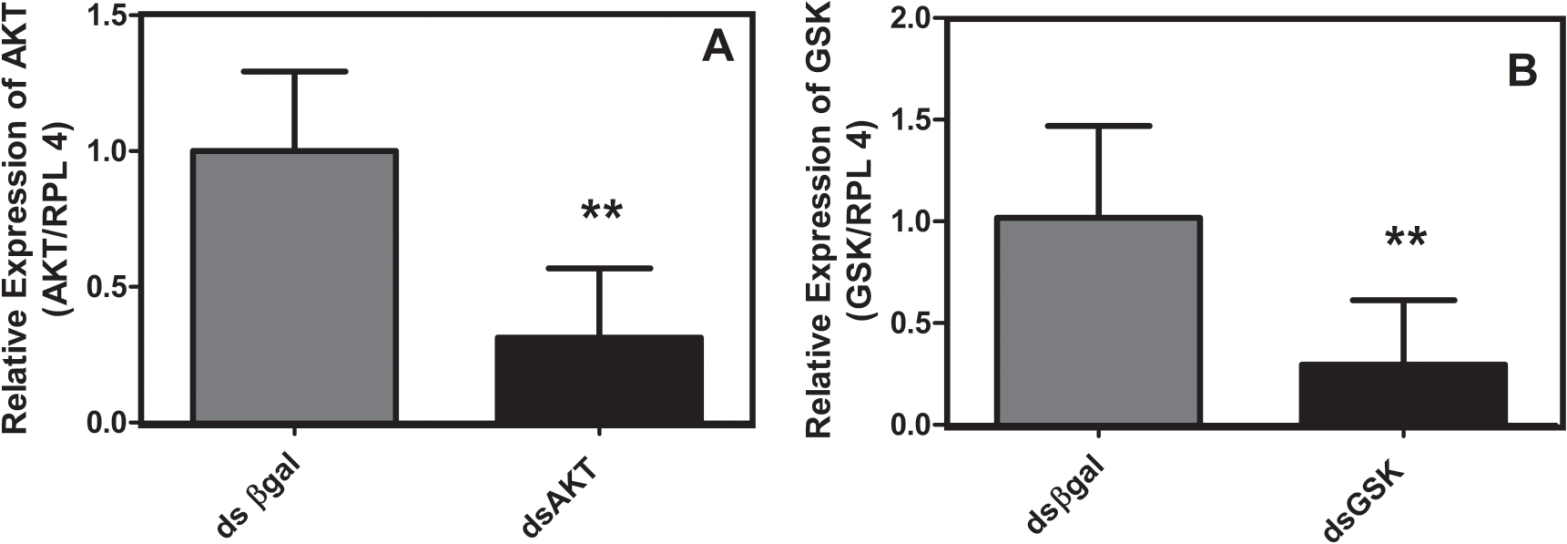
Validation of AKT and GSK3 mRNA knockdown in electroporated *R*. *microplus* eggs. Eggs collected at the stages indicated were de-waxed and electroporated with AKT (A) or GSK (B) dsRNA. Quantitative PCR analyses were performed 7 days later and compared with eggs treated with dsβGal (Refer to Methodology) using primers previously described. Statistical analysis was carried out using the Student t test (*p*<0.05), (Triplicate; n = 3).

To analyze gene silencing, primers were designed outside the sequence used for dsRNA synthesis, as shown in Fig 4. Knockdown was observed for both AKT and GSK genes seven days after egg electroporation. AKT transcript was silenced by approximately 50%, (Fig 4A). While, GSK transcript was silenced by nearly 65% (Fig 4B).

### 3.4 Glycogen content in AKT and GSK silenced eggs

**Fig 5:**
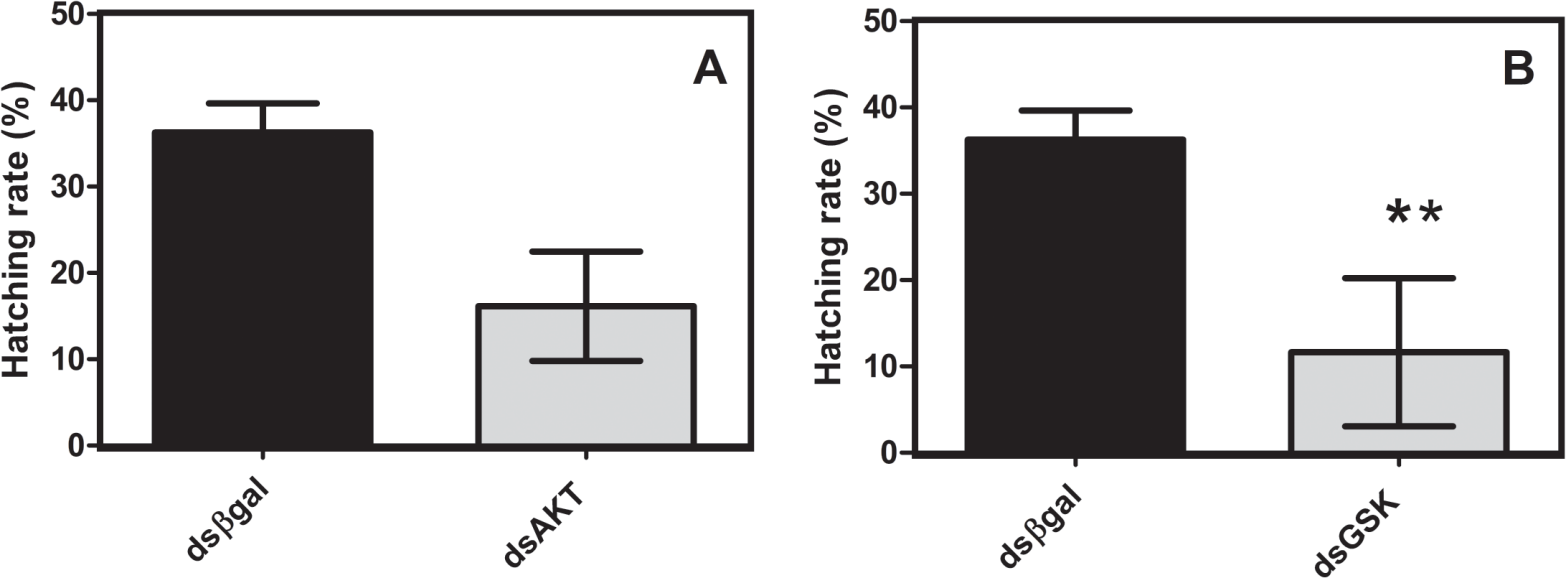
Silencing of AKT and GSK changes glycogen content *R*. *microplus* eggs. Glycogen content was determined in egg homogenates obtained 7 days after electroporation with AKT (A) or GSK (B) dsRNA and compared with eggs treated with βGal dsRNA. Statistical analysis was carried out using the Student t test (*p*<0.05), (Triplicate; n = 3).

When dsAKT was used to electroporate *R*. *microplus* eggs it glycogen decreased levels by 45% (Fig 5A). In eggs electroporated with dsGSK a slight reduction (17%) in glycogen content (Fig 5B).

### 3.5 Hatching rate in AKT and GSK silenced eggs

**Fig 6:**
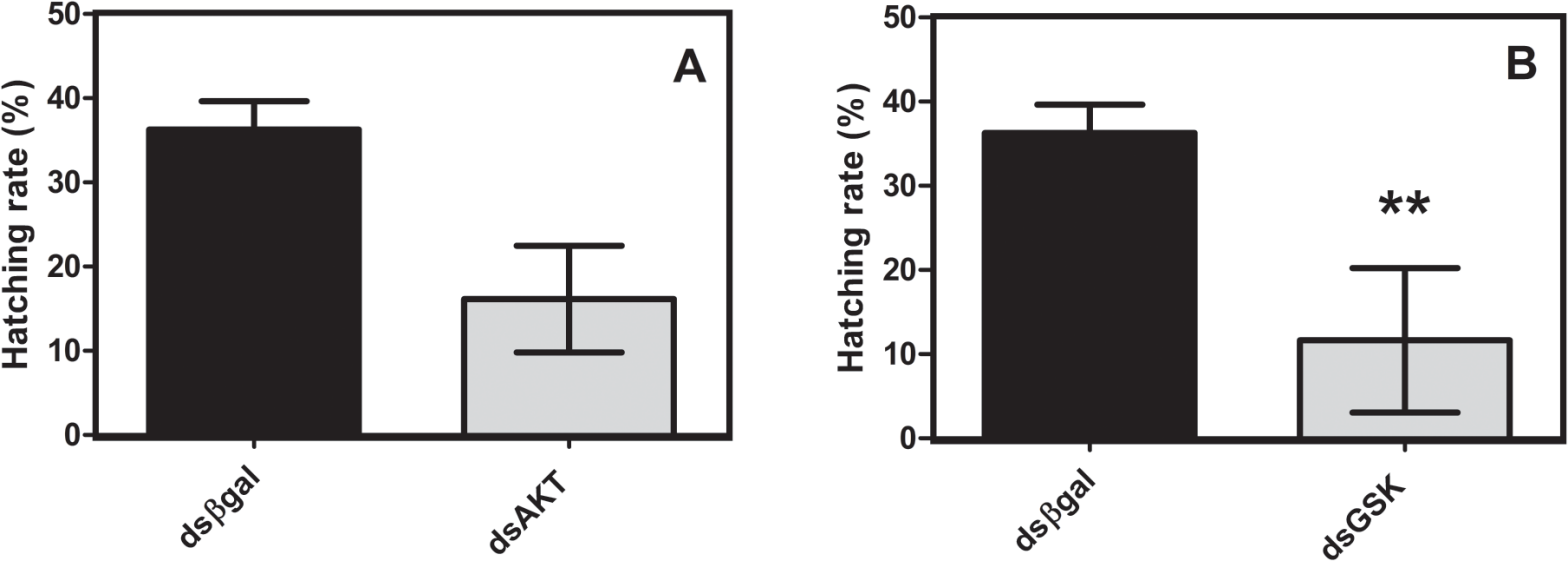
AKT and GSK knockdown inhibits *R*. *microplus* hatching rate. Following electroporation with AKT (A) or GSK (B) dsRNA, de-waxed eggs at the developmental stages indicated were kept at 28° C and 80% relative humidity until hatching. Immediately after hatching, larvae were frozen at -20° C and then subsequently counted as described. Percentage values refer to the number of larvae relative to total number of eggs (in 150 mg aliquots, in replicates). Statistical analysis was carried out using the Student t test (*p*<0.05), (Triplicate; n = 3).

Egg hatching was significantly reduced by 60%, in eggs electroporated with dsGSK (Fig 6), when compared with control treatment.

## Discussion

The RNA interference tool is widely used to study gene function. This method allows specific gene silencing through the synthesis and delivery of double-stranded RNA of cognate target gene. The dsRNA delivery methods include injection, soaking, feeding, electroporation and association with viruses, bacteria, and lipoproteins [4, 18–21]. Delivery of nucleic acids by electroporation has been used in various organisms, such as *Haemonchus contortus*, *Camphylobacter jejuni*, *Bombyx mori* and *Ixodes scapularis* [3, 22–24]. Electroporation to deliver dsRNA can be considered a non-invasive technique, despite causing micro-cracks in the cytoplasmatic membrane, which allow double stranded RNA to enter cells, triggering the RNAi gene-silencing pathway [5].

Allowed us to successfully electroporated these eggs to deliver dsRNA into embryos (Fig 3), attempts to deliver dsRNA to unaltered eggs were unsuccessful (data not shown) a possible explanation is that unaltered eggs did not submerge in dsRNA solution (Fig 1C), and therefore dsRNA by electroporation under the same condition with *R*. *microplus* eggs failed. Here, we have succeeded to deliver dsRNA to de-waxed tick eggs, and to characterize and determine the induced phenotype. After treatment with heptane, the cluster of eggs formed a monolayer (Fig 1A and 1B) that could be properly immersed in a drop containing the dsRNA solution. It enabled (Fig 1D) full contact with the passage of electric current and entry of dsRNA. The electroporation was shown to be an efficient method for delivery of dsRNA into tick eggs. However, tick eggs were shown to be challenging subjects for this technique, since the majority of individual batches were effectively electroporated and repeatability was not easily obtained. Although, there is variation of efficiency of electroporation, in all experiments the electroporated eggs displayed significant reduction in gene transcription and egg viability. We failed to transport Cy3-labeled siRNA through the eggs by electroporation. The same phenomenon was observed in other author [3].

In an effort to shed more light on the effective application of the electroporation delivery of RNAi in ticks, we evaluated two different genes involved in metabolic and embryo development in this organism. The knowledge of embryonic metabolism has been greatly improved in recent years, with a huge contribution of functional-genomic tools and biochemical approaches such as RNA interference. The knowledge obtained from these studies has been successfully applied to uncover different aspects of embryo development from multiple species. Previous reports described the entry of fluorescent-probed dsRNA in different study models [25], including tick eggs and nymphs [2]. Such data help to support the corresponding events derived of RNAi gene silencing on functional-genomic approaches. In the present study, we have built on these earlier successes to show that it is possible to use heptane to remove wax coating from eggs and substantially improve the delivery of dsRNA into tick eggs.

We provide sufficient evidence to show that dsRNA was delivered into embryos, as revealed by re-amplification of electroporated dsRNA using qPCR primers internally to the dsAKT fragment sequence (Fig 3). Additionally, we prove that it is possible to deliver some molecules using electroporation such as DAPI (Fig 2). Suppression of cognate target genes was confirmed using qPCR specific and previously validated [10–11] primers external to AKT dsRNA target sequences, regardless of the developmental stage of the eggs used (Fig 4). Though re-amplification was determined for dsAKT alone (Fig 3B), it is plausible to consider that dsGSK was successfully delivered due to a reduction in its relative expression (Fig 4B) down to values similar to those obtained for AKT dsRNA (Fig 4A). Moreover, molecular and biochemical analyses were performed 7 days after dsRNA delivery by electroporation (Figs 5 and 6).

Our group demonstrated that AKT inhibition, either chemical or through RNAi, reduced both glycogen content and the viability of *R*. *microplus* BME26 embryo cell line, whereas their glycogen content increased after GSK chemical inhibition and RNAi knockdown [10]. Present results show that silencing of either AKT or GSK expression altered embryogenesis, which must be considered when analyzing/interpreting the data from gene silencing studies and these effects depend on the developmental stage gene silencing was performed. Our group was previously observed that AKT and GSK display different relative expression profiles during *R*. *microplus* embryogenesis [11,14].

A previous report on the effects of either GSK silencing or inhibition on *R*. *microplus* egg development and hatching relied on dsRNA injection of adult females [10]. Similar approaches were also used on eggs collected from dsGSK-injected *Tribolium castaneum* [26] and *Aedes fluviatilis* [27] females. In both cases, egg oviposition and hatching were reduced, but glycogen content increased. Moreover, glycogen distribution during *A*. *aegypti* embryogenesis is inversely related with GSK activity [28]. Interestingly, in other models, AKT was described to play different roles in physiological processes during embryo development, such as cell survival, centrosome migration and spindle orientation [29–30]. Further studies are on the way to characterize morphological changes in unhatched eggs.

The treatment of eggs with heptane may have broader and important applications in tick research, since de-waxed and electroporated *R*. *microplus* embryos remained viable and eggs hatched normally (S1 Fig) to perform the analyses herein (Fig 6). Our data, it was clearly shown that: (i) it is possible to improve the delivery of dsRNA into de-waxed tick eggs by electroporation, (ii) delivered dsRNA can trigger suppression of cognate gene mRNA in embryos, (iii) embryogenesis of de-waxed eggs can proceed normally, and (iv) heptane-treated eggs can properly hatch. The effect of gene silencing using dsRNA in embryos was observed in many arthropods by injection or/and feeding [10, 31]. Similar results were reported for *Caenorabditis elegans*, where the effects of RNAi silencing were transtadially transferred up to two generations [32]. Previous studies on *Rhodnius prolixus* demonstrated the persistence of silencing effects on emergence of nymphs [31]. The ability to deliver dsRNA into embryos provides an appealing opportunity to study gene silencing in ticks. In this study, it was revealed that embryo development progressed normally following de-waxing. Conventional strategies to inject dsRNA into females affect the study of isolated effects of zygotic genes silencing and the comprehension of egg specific events, such as the maternal-zygotic transition (MZT). This event is highly conserved among metazoans and comprises two events that occur during early animal embryogenesis: elimination of maternally provided mRNAs and synthesis of new transcripts from zygotic genome [33–35]. Further details of MZT on tick eggs remains to be unraveled. Furthermore, this approach creates new opportunities to determine if cognate target gene suppression on tick embryos is transstadial. In conclusion, the improvement of electroporation described here will be a useful approach for gene silencing in tick eggs, and an important tool for developmental biology in ticks and probably other organisms.

## Supporting Information

S1 Fig The eggs treated with heptane and electroporation can develop and hatched. In columns are the eggs 1^st^, 9^th^ and 15^th^ day of development respectively, the lines are the different treatments performed. Unaltered (Un) treated with heptane (WH) and treated with heptane and electroporated with water (WHE). Magnification bars: 2000 μm.
(TIF)
