# Correction: Non-Invasive Delivery of dsRNA into De-Waxed Tick Eggs by Electroporation

**DOI:** 10.1371/journal.pone.0133949

**Published:** 2015-07-24

**Authors:** Newton Ruiz, Leonardo Araujo de Abreu, Luís Fernando Parizi, Tae Kwon Kim, Albert Mulenga, Gloria Regina Cardoso Braz, Itabajara da Silva Vaz, Carlos Logullo

Fig 5, “Silencing of AKT and GSK changes glycogen content *R*. *microplus* eggs,” is incorrect. Please see the correct Fig 5 and its caption here.

**Fig 5:**
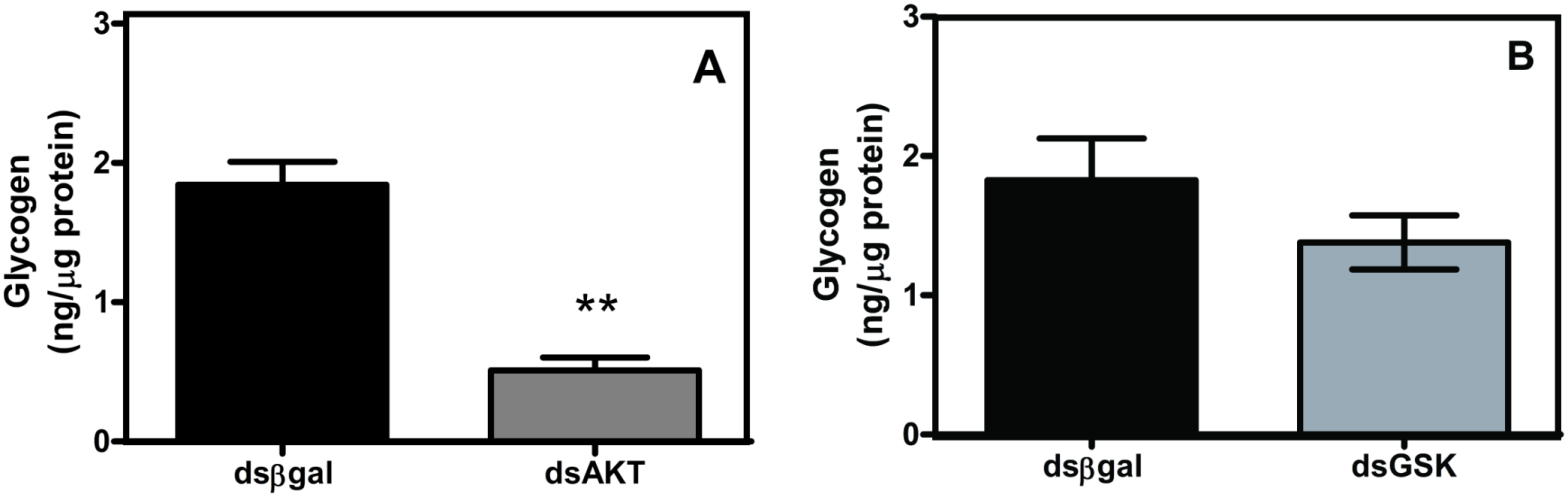
Silencing of AKT and GSK changes glycogen content *R*. *microplus* eggs. Glycogen content was determined in egg homogenates obtained 7 days after electroporation with AKT (A) or GSK (B) dsRNA and compared with eggs treated with βGal dsRNA. Statistical analysis was carried out using the Student t test (p<0.05), (Triplicate; n = 3).
